# Integrating evolution in the management of captive zoo populations

**DOI:** 10.1111/eva.12258

**Published:** 2015-04-17

**Authors:** Albrecht I Schulte-Hostedde, Gabriela F Mastromonaco

**Affiliations:** 1 Department of Biology, Laurentian UniversitySudbury, ON, Canada; 2 Reproductive Physiology, Toronto ZooScarborough, ON, Canada

**Keywords:** adaptation, captive breeding, domestication, immune system, mating system, zoos

## Abstract

Both natural animal populations and those in captivity are subject to evolutionary forces. Evolutionary changes to captive populations may be an important, but poorly understood, factor that can affect the sustainability of these populations. The importance of maintaining the evolutionary integrity of zoo populations, especially those that are used for conservation efforts including reintroductions, is critical for the conservation of biodiversity. Here, we propose that a greater appreciation for an evolutionary perspective may offer important insights that can enhance the reproductive success and health for the sustainability of captive populations. We provide four examples and associated strategies that highlight this approach, including minimizing domestication (i.e., genetic adaptation to captivity), integrating natural mating systems into captive breeding protocols, minimizing the effects of translocation on variation in photoperiodism, and understanding the interplay of parasites/pathogens and inflammation. There are a myriad of other issues that may be important for captive populations, and we conclude that these may often be species specific. Nonetheless, an evolutionary perspective may mitigate some of the challenges currently facing captive populations that are important from a conservation perspective, including their sustainability.

## Introduction

It is axiomatic that all organisms are the product of their evolutionary history. In particular, natural selection is responsible for the adaptations that optimize reproduction and survival of organisms in the environments that they occupy. On the other hand, captive populations are of interest because these populations have typically been removed from the environments that they are adapted for, and which are, in most cases, much more complex than the captive environments. Populations can adapt rapidly to captive environments through domestication, in which humans impose artificial selection in order to increase the prevalence of desired traits in the domesticated population. For domestic animals and plants, human breeders choose to breed only those individuals that thrive in the captive environment, leading to transgenerational changes that result in a population that is adapted to breed and survive in the conditions imposed by the breeder. For example, the American mink (*Neovison vison*) has been farmed since the late 1800s to supplement the high demand for fur. Mink have been bred intensively for artificially selected traits including size and temperament (Belliveau et al. 1999; Kruska and Sidorovich 2003), but especially for a broad range of fur colors, from snow white to jet black (Shackelford 1948; Joergensen 1985).

Among captive populations of animals, zoo populations are unique in that they are maintained to educate the public regarding wildlife and their habitats and/or to preserve critically endangered species through captive breeding and reintroduction programs. Although assessment and preservation of genetic diversity is a top priority for most conservation breeding programs, fundamental to these goals is the maintenance of the genetic variation of these captive populations (Lacy 2009). Whether used to further educational or conservation goals, it is critical that these captive populations are representative of the natural populations from which they are derived. Other authors have advocated for the importance of incorporating evolutionary perspectives in the decision-making process for conservation programs in general (Ashley et al. 2003). However, maintaining captive populations, such that they are reflective of the wild phenotype of the animal, can be challenging in zoos because of the mismatch between the environment that the zoo population is originally from and the captive context in which they are being housed (DeWitt et al. 1998; Hendry et al. 2011; Carroll et al. 2014). For example, solitary animals with large territories that only encounter sexually mature counterparts during estrus may be housed in proximity of their mates year-round, potentially leading to behavioral issues, including sexual aggression or sexual incompatibility. Other stressors can exist in captive environments for which animals are not adapted, including the acoustic environment, physical substrate, and even availability of food (Morgan and Tromborg 2007). Minimizing the mismatch between the natural environment and the captive environment should limit the decline and poor performance of captive populations (DeWitt et al. 1998; Hendry et al. 2011; Carroll et al. 2014).

Populations may match their phenotype to an environment via phenotypic plasticity rather than genetic adaptation. Phenotypic plasticity can lead species to rapidly match the optimal phenotype for the environment being occupied, and thus can allow individuals to overcome pressures exerted by the captive environment, particularly a zoo setting. Altered behaviors, such as a change in temperament (e.g., lack of fear of humans/unknown, decreased foraging, or predatory activity), are routinely observed in captive animals (McDougall et al. 2006). Furthermore, there is evidence that suggests that differences in factors, such as diet, exercise, and psychological stimulation, result in changes in physiology and morphology in captive individuals (O'Regan and Kitchener 2005). Nonetheless, although perhaps important for longer lived species with long generation times and slow rates of evolutionary adaptation, phenotypic plasticity and other nongenetic changes including maternal effects are unlikely to mitigate the consequences of adaptation to a captive environment in the long term (Hendry et al. 2011).

Currently, programs implemented to support rapidly declining species have attempted to address the impact of human-altered environments on the evolutionary processes of species in captivity (Smith and Bernatchez 2007), but it is clear that more attention is needed to deal with the evolutionary consequences of captive breeding for conservation (Frankham 2008; Pelletier et al. 2009). Managers of zoo populations with conservation programs have an inherent interest in maintaining the genetic and evolutionary integrity of the species they administer, and this is reflected in the care and research surrounding issues related to maintaining genetic diversity, including managing reproductive hormone cycles to maximize production of genetically diverse offspring, and associated discussions related to the sustainability of captive breeding of endangered species for subsequent release (e.g., Kleiman et al. 2010; Fa et al. 2011). It is also clear that the captive environment does not always reflect the natural adaptive environment for most species in zoos, but captive animal programs rely on maintaining these species such that they reflect their natural genetic/adaptive state. Thus, an evolutionary perspective on zoo population management may be fruitful in overcoming limitations currently experienced by some captive breeding programs. These limitations include poor reproductive success, leading to increased levels of inbreeding and the concomitant effects on behavior, morphology, and continued reproductive success (inbreeding depression) (Snyder et al. 1996; Lacy 2013).

Here, we propose that zoo populations, particularly those under captive breeding protocols for eventual reintroductions, may benefit from a more explicit consideration of the evolutionary context of captivity beyond the generally stated goal of maximizing genetic diversity of captive populations—that is, we expect that zoo population managers can improve the reproduction and health of captive species by addressing the evolutionary mismatch between the environment that their species are adapted for, and the captive zoo environment in which they currently exist. The zoo community has recognized the need to consider evolutionary perspectives in some aspects of their programs, including inadvertent adaptation to captivity, the need to maximize genetic diversity, and recently the consequences of enforced monogamy in captive breeding protocols. Yet, beyond these important issues, there are further opportunities to consider the role of evolution in captive zoo populations. We briefly review current themes in zoo conservation biology that reflect evolutionary perspectives, and then expand on these by describing new ways that evolution can be integrated into the management of zoo animal reproduction and health.

## Current evolutionary perspectives in zoo population management

### Captive breeding success and unintentional domestication

Genetic adaptation to an artificial environment, under the influence of human selection criteria, is synonymous with domestication. The inadvertent domestication of long-term captive zoo populations has been an area of concern for some time among zoo biologists because of their use for conservation, particularly the reintroduction of endangered species into the wild (Pelletier et al. 2009). Captive breeding programs intensively manage reproduction, population size, and demographics in an effort to control inbreeding and genetic drift (Foose and Ballou 1988; Lacy 2009; Ballou et al. 2010). However, small population sizes can lead to a rapid accumulation of deleterious alleles (Lynch and O'Hely 2001), and the main strategy used to minimize genetic change (equal breeding of founders) is often difficult, if not impossible, with many species in captivity, particularly mammals and birds (Snyder et al. 1996). Of particular note is that many of the large, charismatic megafauna (typically mammals that tend to draw visitors) are necessarily kept at relatively small population sizes because of the practical limitations associated with their husbandry in zoos, and thus they may be more susceptible to factors such as genetic drift. A perusal of studbooks from various captive mammals shows that a single founder often produces a disproportionately higher number of offspring than the remaining founders resulting in a higher genetic contribution to subsequent generations (Table1).

**Table 1:** Sample of studbooks of 14 endangered mammals that indicate unequal contribution of founders to first generation of offspring due to highly productive male and female founders. Only regional studbooks (American Association of Zoos and Aquariums) of species containing less than 60 wild-caught animals were used due to the difficulty in data management. Only original founders were counted (i.e., ones that were used to initiate captive population; not ones that were introduced decades later to refresh the genetics of the captive population). In parentheses are (a) the percentage of individuals that acted as founders (i.e., successfully mated in captivity) of the number of individuals initially taken into captivity, (b) the percentage of total offspring produced by the founders that are attributed to the most successful male/female. Studbook references available as Table S1

Species	No. animals from the wild	No. founders (a, %)	No. founder males	No. of offspring from founder males	No. of offspring from top male (b, %)	No. founder females	No. offspring from founder females	No. of offspring from top founder female (b, %)
Coquerel's sifaka*	24	12 (50)	5	34	19 (55.9)	7	47	12 (25.5)
Gerenuk†	33	21 (63.6)	6	104	33 (31.7)	14	69	10 (14.4)
Indian rhinoceros‡	46	30 (65.2)	14	61	24 (39.3)	16	39	7 (18)
Klipspringer§	30	15 (50)	7	39	20 (51.2)	8	25	13 (52)
Kordofan aoudad¶	20	16 (80)	6	106	58 (54.7)	10	72	15 (20.8)
Lesser kudu**	36	19 (52.8)	9	80	28 (35)	10	48	14 (29.2)
Persian onager††	47	23 (48.9)	10	79	18 (22.7)	13	38	8 (21)
Red river hog‡‡	23	11 (47.8)	7	94	25 (26.6)	4	27	18 (66.7)
Sand cat§§	20	9 (45)	5	63	28 (44.4)	4	54	24 (44.4)
Spectacled bear¶¶	58	27 (46.5)	13	69	18 (26.1)	14	62	17 (27.4)
Speke's gazelle***	10	9 (90)	3	36	34 (94.4)	6	41	15 (36.5)
Spotted necked otter†††	33	12 (36.4)	4	40	22 (55)	8	47	8 (17)
Sumatran tiger‡‡‡	18	16 (88.9)	6	33	22 (66.7)	10	51	22 (43.1)
White-lipped deer§§§	8	8 (100)	3	47	24 (51.1)	5	55	14 (25.5)

* *Propithecus coquereli*.

† *Litocranius walleri*.

‡ *Rhinoceros unicornis*.

§ *Oreotragus oreotragus*.

¶ *Ammotragus lervia blainei*.

** *Tragelaphus imberbis*.

†† *Equus hemonius*.

‡‡ *Potamochoerus porcus*.

§§ *Felis margarita*.

¶¶ *Tremarctos ornatus*.

*** *Gazella spekei*.

††† *Lutra maculicollis*.

‡‡‡ *Panthera tigris sumatrae*.

§§§ *Przewalskium albirostris*.

Captive environments are very different from the wild and can impose different selection pressures that can lead to genetic adaptation to captivity that affects behavior (e.g., temperament; McDougall et al. 2006), morphology (e.g., size, skeletal morphometrics; O'Regan and Kitchener 2005), and reproductive output (e.g., age at sexual maturity, litter size). In particular, populations of species with short generation times will adapt more rapidly to captivity than those with longer generation times (Frankham 2008). Despite an explicit desire to prevent adaptation to captivity (Lacy 2009) and strategies such as ‘hands-off’ rearing techniques, standardization and strict management of food intake, and implementation of species-specific behavioral enrichment, certain individuals often interact better with their keepers and environment and flourish in the captive setting, thereby increasing their genetic contribution to subsequent generations. The types of practices and circumstances that occur in zoos can influence many life history traits, including age at first and last reproduction (Mace and Pelletier 2007). These, and other factors, can inadvertently lead to evolutionary change away from the optimal phenotype of an organism for success in the wild.

These evolutionary changes to the phenotype can pose a significant problem for species reintroduction programs. For example, steelhead trout (*Oncorhynchus mykiss*) that are released after only a single generation in captivity have a significant reduction in reproductive success (Christie et al. 2012). Thus, physiological, morphological, and behavioral attributes that confer success in captivity may be incompatible with success in the wild, and natural populations with a large proportion of reintroduced animals may be unsustainable (Lynch and O'Hely 2001).

How do zoos thwart inadvertent adaptation to the captive environment? If species management plans included systematic collection and cryopreservation of genetic material early in life when sexual maturity is attained (for example during routine health exams), particularly from individuals that are not far removed from the ‘wild’ generation, then reproductive technologies [e.g., cryopreservation of gametes/embryos, artificial insemination (McLaren 2009)] can be applied to more effectively genetically manage captive populations by integrating genetic material that has either not been subjected to, or has undergone less, selection for the captive environment. The relative efficacy of biotechnology to minimize adaptation to captivity has been variable, with sperm far more likely to be cryopreserved than ova, perhaps because oocytes are more sensitive to the disruption that can occur from freezing (Williams and Hoffman 2009). These approaches, particularly if sperm are cryopreserved from wild populations and used to fertilize the eggs of captive females, are no panacea—sperm may not remain viable and larger numbers of sperm are required to fertilize ova, source populations may evolve between when the sperm was initially cryopreserved and fertilization of ova such that released offspring are not matched with the environment they are released in, and outbreeding depression may result if genetic divergence occurs between the source population and the population that is released (Dylan 2008).

Managers may also minimize the effects of inadvertent adaptation by preferentially breeding those individuals that exhibit a ‘wild’ phenotype. Determining which phenotypic traits are most important to maintain will be challenging, but collaboration with researchers that conduct research on the focal or related species may provide useful insight.

### Short-circuiting natural mating systems with captive breeding protocols

One of the fundamental requirements of breeding an endangered species in captivity is to minimize inbreeding. Individuals are typically paired based on mean kinship (the average relatedness of an individual to the population) calculated from a known pedigree (Ballou et al. 2010). To minimize the deleterious effects of inbreeding, especially in the small effective population sizes of zoo populations, managers of captive breeding programs preferentially breed individuals with the fewest close kin in the population. This strategy maximizes genetic diversity, reduces adaptation to captivity, and reduces the loss of genetic variation associated with genetic drift, which can be rapid in small populations (Ballou et al. 2010). The pairing of individuals based on specific criteria (relatedness) imposes monogamy and minimizes or removes sexual selection [the competition for mates by one sex (usually males) and/or discriminating mate choice by the other (usually females)]. The lack of sexual selection integrated into captive breeding programs may explain why the success of captive breeding programs has been mixed, with some programs being deemed unsustainable in part because of failure of some proportion of pairs to reproduce (Snyder et al. 1996; Leus et al. 2011). The mating strategies each sex employs are adaptations to maximize Darwinian fitness and so reproductive failure should not be surprising when instituting a breeding protocol that does not allow aspects of the natural mating system of the species to be performed (e.g., female mate choice, sperm competition). Using knowledge of natural mating systems should help the development of noninvasive techniques to enhance breeding success of captive populations, assisting in the management of these often highly endangered species (Chargé et al. 2014). Indeed, there is ample evidence from studies of both captive and natural populations suggesting that offspring quality is enhanced by sexual selection (e.g., Firman and Simmons 2012; Gerlach et al. 2012; Raveh et al. 2014).

The need for natural behaviors to be expressed by captive wildlife has long been recognized (reviewed in Wielebnowski 1998; McPhee and Carlstead 2010), but only recently has the zoo community recognized that aspects of sexual selection [e.g., mate choice (Swaisgood and Schulte 2010; Asa et al. 2011)] should be considered as a component of captive breeding programs in zoos. In particular, it has been recognized that the genetic benefits of mate choice can improve reproductive outcomes in captive breeding programs (Wedekind 2002). Although there is increasing interest in implementing mate choice and it has been discussed for various zoo-based breeding programs (e.g., Grahn et al. 1998; Bryant 2005; Swaisgood and Schulte 2010; Asa et al. 2011), thus far, there have been no published experimental studies that compare breeding based on pedigree and breeding based on mate choice in a zoo setting. Recent studies, however, have shown that integrating aspects of natural mating systems into supportive or captive breeding programs can enhance the productivity of these programs (e.g., Fisher et al. 2006; Pitcher and Neff 2007).

Given the evidence from aquaculture in which positive outcomes have been acquired by integrating mate choice into captive breeding (Pitcher and Neff 2007), it may be prudent to apply similar protocols to zoo populations (Chargé et al. 2014). Nonetheless, despite the recent interest in mate choice, it is clear that integrating sexual selection into captive breeding programs must consider all aspects of sexual selection. The use of mate choice to enhance outcomes is predicated on the assumption that mate choice is important in the mating system of the focal species. This may or may not be the case and any protocol that is used in which natural mating systems are integrated into captive breeding must have an understanding of what the natural mating system is in the wild. For example, male–male competition in the form of combat or sperm competition may be integral to reproductive success, rather than mate choice. In some cases, field studies of endangered species are not possible, and so zoo managers may collaborate with researchers that study related species to develop experiments to determine whether better outcomes can be achieved than when using traditional captive breeding protocols. Using a mate choice design (e.g., Woodgate et al. 2010) or mixing sperm during artificial insemination to induce sperm competition (e.g., Boschetto et al. 2011) are two of many approaches in which the natural mating system may be integrated into captive breeding. These approaches would need to be balanced with the need to maintain effective population sizes in the face of a potential increase in variation in reproductive success.

## Expanding evolutionary perspectives in zoo population management

Perhaps the greatest challenge faced by zoos and captive breeding programs is the mismatch between the environment in which the focal species has evolved and is adapted for, and the environment provided by the zoo. While the artificiality of the captive environment is readily acknowledged and efforts are made to ensure that it reflects the natural habitat as much as possible (Fa et al. 2011), a broad appreciation beyond the differences in habitat may provide insights that can enhance the health and management of captive zoo populations.

### Melatonin, photoperiod, and changes in latitude

Translocating any species from its native range into captivity has a number of challenges, including the consequences associated with translocating an animal to a different latitude. For example, the housing of polar species in temperate zoos not only leads to these species experiencing a different climate (assuming the species is housed outdoors) but also a different photoperiod. Over the course of a year, polar species are adapted for lengthy periods of complete to near darkness (polar winter) coupled with lengthy periods of light. Tropical species are adapted to equal periods of light and dark over the year. Zoos located in temperate regions are aware of the temperature differences among these latitudes (Fa et al. 2011), but what about the photoperiod of species that are housed outdoors? Photoperiod is tied to a number of hormonally mediated processes, and thus translocation from a locally adapted photoperiod may have effects on reproduction and health.

Reproductive physiology and health can be affected by changes in photoperiod through differences in melatonin levels in mammals. Melatonin is a hormone secreted by the mammalian pineal gland at increased levels in darkness that regulates circadian and circannual rhythms and functions especially as a seasonal clock regulating photoperiod-dependent systems, such as reproduction and behavior (Morgan and Mercer 1994). Changes in the duration of melatonin secretion (i.e., changes in day length) are a passive signal used by the reproductive axis to adjust testicular and ovarian physiology in seasonal animals (Reiter et al. 2009). Melatonin-related seasonality also triggers other physiological and morphological changes, such as coat condition, food intake, and body weight (Morgan and Mercer 1994). Melatonin is involved in the regulation of numerous body functions, and alteration of melatonin levels and, therefore, circadian rhythms (e.g., light at night), has been implicated in breast cancer and mammary tumors in humans and rodents (Stevens and Rea 2001), as well as immune system suppression, depression, and other neurological disorders (Malhotra et al. 2004).

Photoperiod has been identified as an important consideration when housing captive animals in zoos (Asa et al. 2011; Fa et al. 2011), but housing animals outdoors at a different latitude than that from which they have been translocated from can expose animals to photoperiods that they are not adapted for. One consequence of these differences in photoperiod can be variation in melatonin secretion, especially in mammals. Given the role of melatonin in regulating reproductive physiology and other aspects of health, one hypothesis that can be raised is whether examples of reproductive dysfunction found in some species may be the result of this mismatch in melatonin secretion dynamics. We know very little about the natural dynamics of melatonin secretion, whether populations are locally adapted to their photoperiod (and associated melatonin secretion profiles) and how this would be affected if they were exposed to different photoperiods. There is some evidence, however, that photoperiodism (the initiation of reproduction as a result of photoperiod) is a heritable trait, with some variation in the response of some populations (Heideman et al. 1999). This suggests that selection in the captive environment may lead to adaptation to the ‘new’ photoperiod. Some species alter biological processes, such as sexual maturity [white-footed mouse: Carlson et al. 1989; deer mouse (*Peromyscus maniculatus*): Dark et al. 1983] and reproductive seasonality (white-tailed deer (*Odocoileus virginianus*): Bubenik et al. 1990; gray wolf (*Canis lupus*): Mech 2002) across their geographic range, but it is unclear how this degree of flexibility is related to melatonin levels. The zoo literature divides species into long-day and short-day breeders (e.g., Asa et al. 2011), but scant consideration has been made regarding the role of melatonin.

It is clear that we know very little about variation in melatonin secretion and its effects on reproduction in captive zoo populations, but it would not be surprising if melatonin had a role to play in explaining reproductive dysfunction in some species. The degree of phenotypic plasticity in the response to melatonin secretion is also unknown, but we might expect that species with ranges that are limited to a specific latitude may be more susceptible to reproductive dysfunction as a result of translocation to a different latitude because of a higher degree of local adaption than a species with a broad range encompassing a wide latitude. To date, there is no information in the scientific literature regarding the influence of photoperiod on melatonin levels in translocated wildlife species. Alterations in melatonin levels, and therefore, in circadian and circannual rhythms, can result in maladaptive patterns of reproduction and behavior, as well as long-term effects on overall health. Although extensive efforts are made to control lighting for species housed in indoor environments (e.g., timed lighting for rodents, small primates, fishes, reptiles, amphibians, and birds), this can be impossible in species maintained primarily in outdoor enclosures. Monitoring and supplementing melatonin levels may be a possible method for animal managers to regulate circadian and seasonal rhythms of translocated species to enhance reproduction and welfare. Melatonin implants have been used to regulate reproductive performance in sheep (*Ovis aries*) (Hanif and Williams 1991; O'Callaghan et al. 1991; Haresign 1992), deer (*Dama dama*) (Asher et al. 1988), and silver fox (*Vulpes vulpes*) (Forsberg et al. 1990). However, further research is required to develop a better understanding of the impact of changes in latitude on melatonin secretion and the potential benefits of therapeutic melatonin administration to captive breeding programs that are not sustainable.

### Inflammation and the ‘Hygiene Hypothesis’

Disease is a major pre-occupation of captive breeding programs in zoos (Travis and Barbiers 2010), both in terms of zoonotic disease that can be transmitted to humans (Bender and Shulman 2004) and pathogens that can cause disease in the captive animals (Deem 2007). Yet many parasites and pathogens are locked in coevolutionary relationships that can have benefits for their hosts (Zaccone et al. 2008). Eradication of some disease-causing organisms can therefore have unforeseen consequences for the health of captive populations. In addition, there are the conservation implications associated with the eradication of parasites and/or pathogens that have coevolved with their hosts (Jørgensen 2015). Thus, the general approach of limited risk tolerance with respect to captive population husbandry (Miller 2007) may have some important limitations.

The immune system is adapted to protect an individual from pathogens and parasites. Parasites are adapted to evade the defenses of the host immune system, and hosts evolve new defenses to prevent infection leading to coevolution between hosts and parasites. Recently, the prevalence of autoimmune disorders in Western human populations has been attributed to the ‘hygiene hypothesis’ (Okada et al. 2010). This hypothesis posits that the overemphasis on personal and public hygiene in Western society has lead to a rapid shift from the environment which humans are adapted for (the hunter–gatherer environment) to a modern lifestyle that includes intensive medical care. This has lead to an environment devoid of many of the microorganisms and parasites that humans have coevolved with. For example, helminth infections were ubiquitous throughout human evolution, but are now rare in modern Western society (Weinstock and Elliott 2009). There is some suggestion that gastrointestinal disorders, such as inflammatory bowel disease, may be the result of a dysfunctional immune system that is adapted for fighting helminth worms, but which does not encounter them (Rook 2009). The anti-inflammatory properties of helminth worms are now recognized, and therapeutic use of selected species is being developed to treat a variety of inflammatory bowel conditions (Weinstock and Elliott 2009).

Very little is known about the prevalence of autoimmune disorders in natural populations of animals although there is some evidence that self-reactive antibody responses are associated with negative fitness consequences (Graham et al. 2010). Many endangered species in captivity, especially those in breeding programs associated with reintroductions, are medicated, vaccinated, and protected from potential pathogens and parasites in an effort to maximize the outcomes for the captive population and avoid transmission of disease to the wild population. Under these conditions, the immune systems of these species are no longer being exposed to the parasites and pathogens that they are adapted to deal with. Many chronic health conditions, especially those associated with inflammation, may be explained by these kinds of autoimmune effects. Thus, a situation analogous to the ‘hygiene hypothesis’ may be occurring in endangered species housed in captive breeding programs.

Inflammation associated with the immune response and the gastrointestinal system may be one area in which autoimmunity may be involved in captive endangered species. Inflammatory responses can cause damage to underlying tissues, leading to down regulation of the inflammatory cells and cytokines responsible for inducing inflammation (Sears et al. 2011). Chronic inflammation can therefore be a serious health problem leading to a loss of functionality in many tissues (e.g., mucosal tissue, brain, and placenta) (Sears et al. 2011). Captive populations may be at particular risk of chronic inflammation because of the interaction between stress and the immune system (Mason 2010). Glucocorticoids (GC) are steroids that aid an individual to cope with stressors but captivity can alter GC levels, which can therefore have concomitant effects on immunity (Martin et al. 2011). In some species, chronic exposure to GCs, such as may be experienced in captivity (e.g., Fanson et al. 2012), can lead to compromised immune function, including the regulation of inflammation (Martin et al. 2011). Evidence for the role of inflammation in captive endangered species can be found in cheetahs, which are particularly susceptible to gastritis, perhaps due to the presence of the bacteria *Helicobacter* (Terio et al. 2012). Gastric inflammation may also be caused by autoimmune disease mediated by the presence of *Helicobacter* (D'Elios et al. 2004).

Management and care of zoo animals can range from absolute quarantine to less intense management in which animals are exposed to a more ‘natural’ environment including parasites and pathogens. For example, Snyder et al. (1996) identified the necessity for isolation facilities (i.e., reduce exposure of the animals to pathogens present in the wild) when breeding animals for reintroduction programs. Travis and Barbiers (2010) outlined several procedures to prevent disease transmission in captive mammals, including quarantine, integrated pest management programs, and isolation from wild mammals. One consequence of this treatment is that the immune systems of these captive populations may not have the parasites and pathogens to fend off, and thus become dysfunctional. By disrupting the coevolution of between parasite and host, some captive breeding programs of endangered species may be inadvertently promoting autoimmune disease. We suggest that this may be a fruitful avenue of research and that some inflammatory diseases in particular may have an autoimmune component.

## Conclusion

There will always be challenges in maintaining wild populations in captivity. These are complicated by the need to maintain wild phenotypes in captive populations to facilitate eventual reintroduction and promote education. We have outlined a number of issues in the context of the evolutionary ecology of captive populations that could be considered when maintaining these populations, but this is by no means an exhaustive list. For example, the relatively recent understanding of epigenetics has led to the realization that nongenetic changes can result in rapid adaptation to novel environments (Bonduriansky et al. 2012). Wild populations placed in captive environments may thus be at risk of rapid adaptation via this mechanism. Other issues include the recent observation that the release of captive-bred endangered species can lead to the transfer of pathogens that have evolved clinical antibiotic resistance (Power et al. 2013).

Captive breeding of endangered species serves a critical function for conservation and education. The sustainability of these efforts has recently been questioned (Lacy 2013), and while there are a number of success stories (e.g., black-footed ferret, Arabian oryx, California condor), zoos continue to be faced with challenges in maintaining populations of endangered species in captivity. Integrating evolutionary perspectives into management protocols for such species may provide an opportunity to enhance success. Developing mitigation strategies to deal with some of the issues raised in this commentary will be challenging and likely species specific. For example, integrating mate choice into breeding programs is predicated on the assumption that the mating system of the focal species involves mate choice rather than sperm competition or other mechanisms of sexual selection. Thus, an understanding of the evolutionary and behavioral ecology of these species is critical to ensure appropriate management plans.

Management of captive populations has progressed significantly since the establishment of standardized breeding and management protocols. Efforts to improve captive breeding outcomes using mean kinship strategies have been successful in a number of species. However, recent concerns over the sustainability of captive populations identify the need to consider changes in animal management plans. Evaluation and implementation of management techniques in which evolutionary aspects of the species are taken into consideration has the potential to enhance success rates and overcome current challenges in captive breeding. Zoo managers may want to consider collaborative relationships with evolutionary biologists and ecologists to facilitate the development of evolutionarily enlightened management plans.

## Supporting Information

**Table S1.** Website: Association of Zoos and Aquariums/Animal Care & Management/Animal Programs.
